# An Enhanced Hybrid Visual–Inertial Odometry System for Indoor Mobile Robot

**DOI:** 10.3390/s22082930

**Published:** 2022-04-11

**Authors:** Yanjie Liu, Changsen Zhao, Meixuan Ren

**Affiliations:** State Key Laboratory of Robotics and System, Harbin Institute of Technology, Harbin 150001, China; changsenz@stu.hit.edu.cn (C.Z.); 22b308008@stu.hit.edu.cn (M.R.)

**Keywords:** mobile robot, visual–inertial odometry, IMU pre-integration, wheel odometry, Helmert variance component estimation

## Abstract

As mobile robots are being widely used, accurate localization of the robot counts for the system. Compared with position systems with a single sensor, multi-sensor fusion systems provide better performance and increase the accuracy and robustness. At present, camera and IMU (Inertial Measurement Unit) fusion positioning is extensively studied and many representative Visual–Inertial Odometry (VIO) systems have been produced. Multi-State Constraint Kalman Filter (MSCKF), one of the tightly coupled filtering methods, is characterized by high accuracy and low computational load among typical VIO methods. In the general framework, IMU information is not used after predicting the state and covariance propagation. In this article, we proposed a framework which introduce IMU pre-integration result into MSCKF framework as observation information to improve the system positioning accuracy. Additionally, the system uses the Helmert variance component estimation (HVCE) method to adjust the weight between feature points and pre-integration to further improve the positioning accuracy. Similarly, this article uses the wheel odometer information of the mobile robot to perform zero speed detection, zero-speed update, and pre-integration update to enhance the positioning accuracy of the system. Finally, after experiments carried out in Gazebo simulation environment, public dataset and real scenarios, it is proved that the proposed algorithm has better accuracy results while ensuring real-time performance than existing mainstream algorithms.

## 1. Introduction

Simultaneous Localization and Mapping (SLAM) technology has rapidly developed recently, and it is widely used in fields such as drones, Augmented Reality (AR), and unmanned vehicles. Because of the low price of vision sensors, the improvement of computing power, and the advancement of algorithms, vision SLAM has received widespread attention [1,2,3] and produced many excellent results. However, poor lighting conditions, fast motion, and lack of texture which prone to large positioning error or even system crashes remain many flaws to pure vision SLAM. In order to improve the robustness and positioning accuracy of the system, VI-SLAM fade in researcher’s sight as a fusion solution of visual measurement and IMU measurement.

The current fusion framework of vision and inertial navigation can be divided into two ways, one is based on filtering, and the other is based on optimization. The optimization-based method tends to achieve more accurate result by transforming the estimation problem into a nonlinear least squares optimization problem which is a bundle adjustment problem, and iteratively solving it to obtain higher accuracy. However, it was not until the sparsity of the Hessian matrix involved in the solution process was discovered that real-time algorithms were developed to overcome large amount of calculation. For example OKVIS [4], VINS [5], and ORB-SLAM3 [6] use a tightly coupled method to simultaneously optimize the visual projection constraints and IMU pre-integration constraints. In order to balance the calculation load and positioning accuracy, they use a sliding window method to maintain a certain number of optimization variables. Although the use of sparse matrix factorization can reduce the amount of calculation, VIO system based on optimization method still occasionally leads to a decrease in the performance of the entire system while the calculation resource are limited because of the necessary occupation of the other modules in the mobile robot system, such as the navigation module which needs to occupy certain system resources in addition to the localization module [7].

To make sure of the real-time capability, the fusion framework was mostly based on the filtering method. The state vector of general EKF-SLAM [8] contains IMU pose and map feature points. While IMU and image information are obtained, state variables are propagated and updated. The accurate estimation of the map points can obtain the estimation of unbiased pose. As one of the typical filtering method, EKF-SLAM still has great potential for further optimization of the computation load because the state vector contains a large number of spatial feature points. Mourikis and Roumeliotis [9] improved the system performance by proposing the Multi-State Constraint Kalman Filter (MSCKF) algorithm, whose state vector does not contain spatial feature points but only the pose of camera or IMU at a limited time in the sliding window. MSCKF can achieve accuracy comparable to optimization-based methods under a small computational load. Subsequently, many improved versions appeared, such as S-MSCKF-VIO [10], which extended MSCKF to use stereo cameras to acquire certain scale information and improve the robustness of the system. ROVIO [11] used luminosity for status update and zero-speed detection, which is fast but has poor accuracy. Li and Mourikis [12,13], whose work was named as MSCKF2.0, added the stable tracking SLAM feature points, the camera-imu external parameters, and the time drift between camera-imu into the state vector, and simultaneously estimated them together with the camera state. Furthermore, they also used the First Estimate Jacobian (FEJ) technology to maintain system observability. OpenVINS [14] is a framework based on the MSCKF sliding window, using a suitable type-based state modular programming method, which facilitates the management of covariance. It integrates various improved methods for MSCKF, such as monocular, binocular, FEJ, SLAM feature points, camera intrinsic and extrinsic parameters estimation, etc., which facilitate the comparison of effects between various improved methods and provides complete theory and programming documentation. Adding a sliding window-based optimization method on the basis of MSCKF framework to provide constraints between the pose states in the sliding window is proposed in [15], but this undoubtedly increases the computational load due to the additional use of optimization methods.

Inspired by optimization-based methods, we can use the IMU pre-integration between two images [16,17] as an observation to update the camera state in the state vector. The idea is similar to one described in [15], but it can greatly reduce the computational load and ensure the real-time performance. Since reprojection error of feature points is also involved in the update process, how to choose a suitable covariance weight between it and IMU pre-integration is very important. HVCE [18] can determine the weights of different types of observations, and has achieved good results in fusion positioning applications in global satellite navigation [19,20]. Xu et al. [21] used the HVCE method to calculate the weights of the point and line factors in the point–line VIO system for proper estimation, which improved the accuracy of the VIO system. The proposed method in this article also use HVCE method considering its effectiveness in multi-observation fusion. 

Hesch et al. [22] analyzed the observability of the VIO system, and concluded that there are four unobservable directions in the VIO system, namely the yaw angle and three position information. Since the linearization point changes during the state update process make yaw become observable from an unobservable state, the paper proposes to use FEJ to maintain the observability of the system and improve the positioning accuracy of the system. In addition, Wu et al. [23] proposed that in some special situations of the robot, VIO will produce additional unobservable directions. For example, in uniform motion, the scale of the system becomes unobservable due to the lack of output acceleration. Quan et al. [24] designed a tightly coupled VIO system for indoor mobile robots by tightly coupling the wheel odometer and gyroscope.

The goal of this paper is to develop a visual–inertial navigation positioning system with high accuracy and low computational load suitable for indoor mobile robots. From this introduction, we can conclude that the optimization-based VIO method can obtain higher positioning accuracy but heavier computation load than the EKF-based VIO method. Currently, VIO systems based on the EKF framework can achieve localization accuracy comparable to optimization-based methods with a smaller computational load. However, from the knowledge we have obtained so far, the mainstream VIO systems based on the EFK framework mostly use IMU measurement for state prediction and covariance propagation but not next update stage. This article proposed a new idea of applying IMU data in system update for further improvement of accuracy. The main contributions of this paper are:

(1) Cosidering the insufficient use of the IMU information in the traditional MSCKF VIO system, the IMU pre-integration method is used to constrain and update the state of the sliding window to improve the positioning accuracy of the system. In order to select the appropriate weight between the covariance of the visual feature point reprojection and the IMU pre-integration, this paper uses the Helmert variance component estimation method in the sliding window update process to select the maximum posterior weights between the visual reprojection and the IMU pre-integration.
(2) For indoor mobile robots in the process of positioning using the MSCKF-based VIO system, there are observable changes (such as loss of scale) during start–stop and uniform motion, resulting in a decrease in positioning accuracy. The speed information provided by the wheel odometer is used for zero-velocity detection, wheel odometer pre-integration, and corresponding status updates to improve the positioning accuracy of the mobile robot system.
(3) Tests and experiments were carried out in the Gazebo simulation environment, public dataset EuRoc [25], and actual environment. The results of tests and experiments were compared with related mainstream algorithms S-MSCKF [10], VINS-Fusion [5,26], and OpenVINS [14]. Simulations and experiments show that the algorithm proposed in this paper can not only ensure real-time performance but also improve the positioning accuracy significantly.

The organization of this paper follows. After the comprehensive introduction in Section 1, the system overview and mathematical method are introduced in Section 2. In Section 3, we build a simulation environment in Gazebo and some simulations are carried out. Next, experimental verification based on the EuRoc data set and the real Mir robot is shown in Section 4. Finally, discussions and conclusions are given in Section 5 and Section 6.

## 2. System Overview and Methodology

### 2.1. System Overview

**Figure 1:**
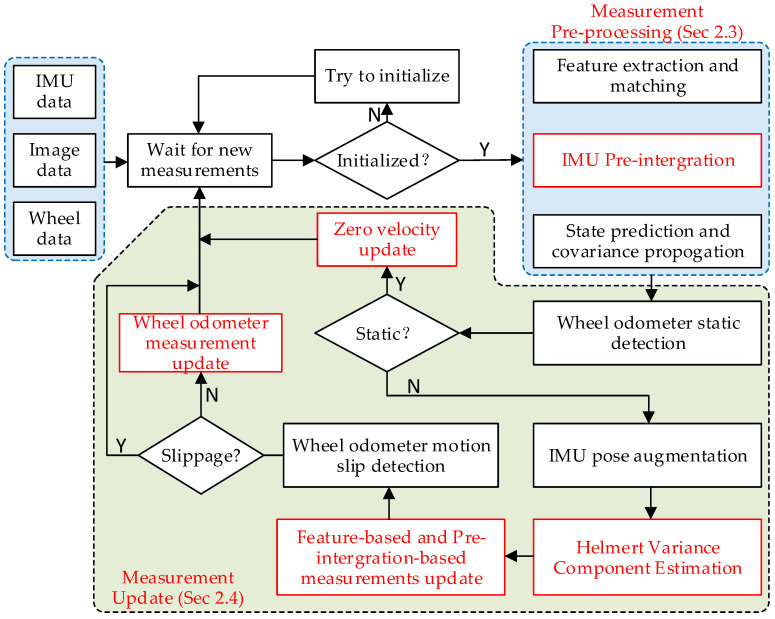
The system framework.

General VIO systems based on MSCKF framework can roughly be divided into two processes. One is to use the measurement of IMU to predict the state and propagate the covariance. The other one is extracting and tracking the visual features of the images from the camera and updating the state by visual geometric constraints. This paper adds the IMU pre-integration module on the basis of the original framework and uses the HVCE method to estimate the maximum posterior covariance weight of the visual constraint and the pre-integration constraint, which are used to simultaneously update the state vector variables in the sliding window. Meanwhile, for the indoor mobile robot, more accurate speed information provided by the wheel odometer is used to detect the zero velocity, and the pre-integration constraint of the wheel odometer is also used to update the pose state. Figure 1 shows the pipeline of the system, in which the red box is the module added in this paper on the basis of the general MSCKF framework.

### 2.2. State Representation

In order to consider accuracy and efficiency, MSCKF framework uses a sliding window algorithm for back-end optimization. The state vector variables include the current IMU inertial navigation state, *N* historical clone camera states, *M* stable SLAM features, time offset between IMU and camera, and the intrinsic and extrinsic parameters of the camera. It is defined as follows:

(1) xk=(xITxCTxLTxWTtIC)xIT=(GIkqTpGIkTvGIkTbωkTbakT)xCT=(GIk−1qTpGIk−1TvGIk−1Tbωk−1Tbak−1T⋯GIk−cqTpGIk−cTvGIk−cTbωk−cTbak−cT)xLT=(pGf1T⋯pGfmT)xWT=(C1IqTpCI0Tς0TC1IqTpCI1Tς1T)

where $x_{I}^{}$ is the state of inertial navigation system (INS) at the moment *k*, and its rotation posture represents the transformation from the global coordinate frame *G* to the local coordinate frame *I_k_* through the unit quaternion ${}_{G}^{I_{k}}q$; pIkG and vIkG represent the position and velocity of the IMU body coordinate frame *I_k_* relative to the global coordinate frame *G*; $b_{\omega_{k}}^{}$ and $b_{a_{k}}^{}$ represent the bias of the gyroscope and accelerometer; $x_{C}^{T}$ is a set of *N* history IMU state clones and, in order to update the pre-integration, it also contains velocity and bias information in addition to the general pose information; $x_{L}^{}$ represents the SLAM features for stable tracking; and the intrinsic camera parameters and the extrinsic parameters between IMU and camera are represented by $x_{W}^{}$.

### 2.3. IMU Dynamic Model and Pre-Integration

A six-axis IMU can measure acceleration and angular velocity of the body frame with respect to the inertial frame through a 3-axis gyroscope and a 3-axis accelerometer, respectively [17]. Its measurement model follows:

(2) ωm(t)=ω^(t)+bω(t)+nω(t)amI(t)=a^(t)+R(qGI)gG+ba(t)+na(t)

where qGI is the quaternion representation from the world frame *G* to the inertial frame *I*, and R(qGI) represents the conversion of the quaternion qGI into the corresponding rotation matrix. The true values measured by IMU are $\hat{\omega }(t)$ and $\hat{a}(t)$, and the measured values are $\omega_{m}(t)$ and amI(t). The kinetic equation is shown as below:

(3) p˙ItG(t)=vItG(t)v˙ItG(t)=aItG(t)q˙GIt=[12ωIt0]⊗qGItb˙ω(t)=nωbb˙a(t)=nab

According to the previous definition, the state vector of INS at time *t* is:

(4) xItT=(GItqTpItTGvItTGbωtTbatT)1×16

Since the use of the real IMU state leads to the singularity of the covariance matrix, there are additional units constrained to the quaternion in the state vector. Therefore, the error IMU state is usually used, which is defined as:

(5) $$\delta x_{I_{t}}^{T}={\left(\begin{array}{ccccc} \delta_{G}^{I_{t}}\theta^{T} & \delta^{G}p_{I_{t}}^{T} & \delta^{G}v_{I_{t}}^{T} & \delta b_{\omega_{t}}^{T} & \delta b_{a_{t}}^{T} \end{array}\right)}_{1\times 15}$$

Assuming the error of the quaternion is $\delta_{G}^{I_{t}}\theta$, the update method is:

(6) qGI←[12δGItθ1]⊗qGI

For vector variable position, speed, and offset, standard update methods can be used (e.g., pIG←pIG+δpIG). Forward integration can be used to calculate the IMU state at time *k+1* from the IMU state at time *k* according to the dynamic equation through the discrete linear acceleration amIk,amIk+1 and angular velocity ωmIk,ωmIk+1 measured by IMU at time *k* and *k* + 1. At the same time, the covariance propagation of the state vector can be performed by linearizing the nonlinear model. According to [14], the error transfer equation can be written in a compact form as:

(7) $$P_{k+1|k}=\Phi_{k}P_{k|k}\Phi_{k}^{T}+G_{k}Q_{d}G_{k}^{T}$$

where *P* is the covariance matrix; $\Phi_{k}$ and $G_{k}$ are the Jacobian matrix of system state and noise characterizing the error transfer; $Q_{d}$ is the discrete noise matrix. For details, please refer to [14]. Referring to [5], the pre-integration from time *i* to *j* can be calculated whose form is defined as follows:

(8) αIjIi=∬t∈[i,j]qbibtaItδt2βIjIi=∫t∈[i,j]qbibtaItδtqIiIj=∫t∈[i,j]([12ωIt0]⊗qIiIt)δt

The formula is the calculation of pre-integration under continuous conditions. The IMU measurements at time *k* and *k* + 1 can be used to accomplish the calculation in the discrete case by numerical methods (such as Euler integral, median integral, or Runge–Kutta method). While considering the bias and noise, the equation can be get as shown:

(9) ω^=12((ωm+nω(k)−bω(k))+(ωm+nω(k+1)−bω(k+1)))a^=12(R(q^bkIi)(amIk+na(k)−ba(k))+R(q^Ik+1Ii)(amIk+1+na(k+1)−ba(k+1)))q^IiIk+1=[12ω^Δt0]⊗q^IiIkα^Ik+1Ii=α^Ik+1Ii+β^IkIiΔt+12a^Δt2β^Ik+1Ii=β^Ik+1Ii+a^Δt

At the beginning of the pre-integration calculation, q^IiIi=[0001]T, α^bk+1Ii, and β^bk+1Ii are zero vectors. It is assumed that the bias is constant in the calculation process. Refer to Appendix A for the calculation method of pre-integration covariance $\Sigma_{ij}$. After calculating the observations q^IiIj, α^IjIi, β^IjIi, and their corresponding covariance $\Sigma_{ij}$, the standard extended Kalman filter can be used to update the state in the sliding window. Note that ${\hat{q}}_{b_{k+1}b_{i}}$, ${\hat{\alpha }}_{b_{i}b_{k+1}}$, and ${\hat{\beta }}_{b_{i}b_{k+1}}$ are affected by the biases $b_{k}^{a}$ and $b_{k}^{g}$, and the bias changes are small. In order to avoid calculating the pre-integration again after the bias is updated, the first-order approximation formula is used directly to update after a small change in the bias:

(10) $$\begin{array}{l} {\hat{q}}_{b_{j}b_{i}}\leftarrow \left[\begin{array}{c} \frac{1}{2}J_{b_{k}^{g}}^{q}\delta b_{k}^{g}\\ 1 \end{array}\right]⊗{\hat{q}}_{b_{j}b_{i}}\\ {\hat{\alpha }}_{b_{i}b_{j}}\leftarrow {\hat{\alpha }}_{b_{i}b_{j}}+J_{b_{k}^{g}}^{\alpha }\delta b_{k}^{g}+J_{b_{k}^{a}}^{\alpha }\delta b_{k}^{a}\\ {\hat{\beta }}_{b_{i}b_{j}}\leftarrow {\hat{\beta }}_{b_{i}b_{j}}+J_{b_{k}^{g}}^{\beta }\delta b_{k}^{g}+J_{b_{k}^{a}}^{\beta }\delta b_{k}^{a} \end{array}$$

where $J_{b_{k}^{g}}^{q}$, $J_{b_{k}^{g}}^{\alpha }$, $J_{b_{k}^{a}}^{\alpha }$, $J_{b_{k}^{g}}^{\beta }$, and $J_{b_{k}^{a}}^{\beta }$ are the pre-integrated Jacobian with respect to the bias, which can be solved iteratively according to the method described in Appendix A.

### 2.4. Measurements Update

#### 2.4.1. Point Feature Measurement Update

The feature points used for visual observation update have two types: SLAM feature points for long-term stable tracking, the state of which can be added to the state vector of the sliding window, and the MSCKF feature points lost in tracking. The predicted pixel coordinates of the spatial feature in the camera image at time *k* can be expressed as:

(11) $$z_{m,k}=h_{d}(h_{p}(h_{t}(h_{r}(\lambda ,\cdots ),R_{C_{k}w},p_{wC_{k}})),\zeta )+n_{k}$$

where $h_{d}(\cdot )$ maps the normalized coordinates to the distorted pixels coordinates; $h_{p}(\cdot )$ converts the coordinates in the camera coordinate frame into normalized coordinates frame; $h_{t}(\cdot )$ maps the 3D coordinates of the feature point in the world frame to the coordinates in the camera coordinate frame, and $h_{r}(\cdot )$ converts the feature point representation λ into 3D coordinates in the world frame. ζ are the camera’s intrinsic parameters, including focal length and distortion parameters. $R_{C_{k}w}$ and $p_{wC_{k}}$ are the position and orientation of the camera; $n_{k}$ is the measurement noise, usually assumed to be Gaussian white noise of one pixel. By stacking the multiple observations of different feature points, the observation equation can be constructed:

(12) rf,k=zm,k−z^m,k=HxδXk+HfδpGf+nk

where $\delta X_{k}$ is the error state vector in the sliding window involved in the feature point observation update; $H_{x}$ and $H_{f}$ are the measurement Jacobian matrix with respect to state vector variables and the position of the feature. For the SLAM feature point update, since the feature point state is included in the state vector of the system, there is no HfδpGf term. As for the update of MSCKF feature points, since the state of the feature points is affected by the state of the camera, the observation equation can be projected onto the left null-space of $H_{f}$ and then updated with a standard extended Kalman filter.

#### 2.4.2. Pre-Integration Measurement Update

The pre-integration of velocity and displacement can be expressed as:

(13) $$\begin{array}{l} \alpha_{b_{i}b_{j}}=q_{b_{i}w}(p_{wb_{j}}-p_{wb_{i}}-v_{i}^{w}\Delta t+\frac{1}{2}g^{w}\Delta t^{2})\\ \beta_{b_{i}b_{j}}=q_{b_{i}w}(v_{j}^{w}-v_{i}^{w}+g^{w}\Delta t) \end{array}$$

The corresponding Jacobian matrix can be obtained:

(14) $$\begin{array}{l} \frac{\partial \alpha_{b_{i}b_{j}}}{\partial \theta_{ig}}={\left[R_{b_{i}w}(p_{wb_{j}}-p_{wb_{i}}-v_{i}^{w}\Delta t+\frac{1}{2}g^{w}\Delta t^{2})\right]}_{\times }\\ \frac{\partial \alpha_{b_{i}b_{j}}}{\partial p_{wb_{j}}}=R_{b_{i}w},\frac{\partial \alpha_{b_{i}b_{j}}}{\partial p_{wb_{i}}}=-R_{b_{i}w},\frac{\partial \alpha_{b_{i}b_{j}}}{\partial v_{i}^{w}}=R_{b_{i}w}\Delta t\\ \frac{\partial \beta_{b_{i}b_{j}}}{\partial \theta_{ig}}={\left[R_{b_{i}w}(v_{j}^{w}-v_{i}^{w}+g^{w}\Delta t)\right]}_{\times }\\ \frac{\partial \beta_{b_{i}b_{j}}}{\partial v_{wb_{j}}}=R_{b_{i}w},\frac{\partial \beta_{b_{i}b_{j}}}{\partial v_{wb_{i}}}=-R_{b_{i}w} \end{array}$$

For quaternions, suppose the ideal observation is:

(15) $$z_{q}={\left[q_{b_{j}b_{i}}⊗(q_{b_{i}w}⊗q_{wb_{j}})\right]}_{1:3}={\left[0\;0\;0\right]}^{T}$$

The residual of the quaternion can then be defined as $r_{q}=z_{q}-{\hat{z}}_{q}$, where ${\hat{z}}_{q}={\left[{\hat{q}}_{b_{j}b_{i}}⊗({\hat{q}}_{b_{i}w}⊗{\hat{q}}_{wb_{j}})\right]}_{1:3}$. The corresponding Jacobian matrix is:

(16) $$\begin{array}{l} \frac{\partial z_{q}}{\partial \theta_{b_{i}w}}=-R_{L}(q_{b_{j}b_{i}})R_{R}(q_{b_{i}w}⊗q_{wb_{j}})\\ \frac{\partial z_{q}}{\partial \theta_{b_{j}w}}=-R_{L}(q_{b_{j}b_{i}}⊗q_{b_{i}w}⊗q_{wb_{j}}) \end{array}$$

where $R_{L}$ and $R_{R}$ are the left and right quaternion multiplication matrices. The residual of pre-integration can be expressed as:

(17) $$r_{I}=\left[\begin{array}{l} r_{q}\\ r_{p}\\ r_{v}\\ r_{b_{g}}\\ r_{b_{a}} \end{array}\right]=\left[\begin{array}{c} -{\left[q_{b_{i}b_{j}}⊗(q_{b_{i}w}⊗q_{wb_{j}})\right]}_{1:3}\\ \alpha_{b_{i}b_{j}}-q_{b_{i}w}(p_{wb_{j}}-p_{wb_{i}}-v_{i}^{w}\Delta t+\frac{1}{2}g^{w}\Delta t^{2})\\ \beta_{b_{i}b_{j}}-q_{b_{i}w}(v_{j}^{w}-v_{i}^{w}+g^{w}\Delta t)\\ -(b_{i}^{\omega }-b_{j}^{\omega })\\ -(b_{i}^{a}-b_{j}^{a}) \end{array}\right]=H_{I}\delta X_{I}+R_{I}$$

where $H_{I}$ is the Jacobian matrix of the pre-integration relative to state in the slide window and $R_{I}$ is the covariance of the pre-integration.

#### 2.4.3. Wheel Odometer Measurement Update

When the wheel odometer detects that the robot’s velocity is zero, it can be defined that the observation speed, acceleration, and angular velocity are all zero. The residual can then be defined as:

(18) $$\begin{array}{l} r_{v}=z_{v}-{\hat{z}}_{v}=-v^{w}\\ r_{a}=z_{a}-{\hat{z}}_{a}=-(\tilde{a}-b_{a}-R_{bg}g)\\ r_{\omega }=z_{\omega }-{\hat{z}}_{\omega }=-(\tilde{\omega }-b_{g}) \end{array}$$

The corresponding Jacobian is:

(19) $$\frac{\partial z_{v}}{\partial v}=I_{3\times 3},\frac{\partial z_{a}}{\partial \theta_{bw}}=-{\left[R_{bw}g\right]}_{\times },\frac{\partial z_{\omega }}{\partial b_{a}}=\frac{\partial z_{\omega }}{\partial b_{g}}=-I_{3\times 3}$$

If the system detects that the robot is in a stationary state, it will not update the visual observation feature points and pre-integration after completing the zero-velocity update. When the speed of the robot is detected by the wheel odometer to be nonzero, $d_{r}$ and $d_{l}$ are the moving distance of the right and left wheels of the differential drive wheel, respectively; $d_{r}$ and $d_{l}$ satisfy [27]:

(20) $$\begin{array}{l} d_{r}=\varepsilon_{r}\cdot \Delta d_{r}+\delta_{r}\sim N(0,\left\|K\cdot \varepsilon_{r}\cdot \Delta d_{r}\right\|)\\ d_{l}=\varepsilon_{l}\cdot \Delta d_{l}+\delta_{l}\sim N(0,\left\|K\cdot \varepsilon_{l}\cdot \Delta d_{l}\right\|) \end{array}$$

where $\Delta d_{r}$ and $\Delta d_{l}$ are the displacement in unit tick of the left and right wheel odometer, respectively; $\varepsilon_{r}$ and $\varepsilon_{l}$ are the scale coefficients; $\delta_{r}$ and $\delta_{l}$ are zero-mean Gaussian distributions whose variance is proportional to the moving distance of the left and right wheels and the scale factor *K*. According to kinematics, the following formula can be obtained:

(21) Δbksbk+1bk=dr+dl2θbkbk+1bk=dr−dld

where *d* is the center distance between the two wheels. Since the calculated result of Formula (21) is one-dimensional, it needs to be expanded into a three-dimensional vector when performing the following calculation, that is: Δbksbk+1bk←(Δbksbk+1bk,0,0) and θbkbk+1bk←(0,0,θbkbk+1bk). The rotation and displacement increments $R_{b_{j}b_{i}}$ and $p_{b_{i}b_{j}}$ of the wheel odometer between image frames *i* and *j* can then be obtained by numerical integration of the dynamic Equation (22); the initial condition $R_{b_{i}b_{i}}$ is identity matrix and $p_{b_{i}b_{i}}$ is a zero vector:

(22) pbibk+1=pbibk+RbkbiTRbkbk’Δbksbk+1bkRbk+1bi=Rbk+1bkRbkbi

where $R_{b_{k}b_{k^{’}}}$ represents the rotation transformation of the intermediate time between *k* and *k+1* relative to the time *k*, which can be calculated by θbkbk+1bk. For the orientation error propagation using **SO**(3) perturbation, we obtain:

(23) $${\tilde{\theta }}_{b_{k+1}b_{i}}\approx {\hat{R}}_{b_{k+1}b_{k}}({\tilde{\theta }}_{b_{k}b_{i}}+J_{r}(-\frac{d_{r}-d_{l}}{d})\frac{n_{r}-n_{l}}{d})$$

where superscript ^ represents the true value and superscript ~ represents the perturbation. $J_{r}(\cdot )$ is the right Jacobian of **SO**(3) that maps the variation in rotation angle in the parameter vector space into the variation in the tangent vector space to the manifold. Then, the error transfer coefficients can be obtained as:

(24) $$\frac{\partial {\tilde{\theta }}_{b_{k+1}b_{i}}}{\partial {\tilde{\theta }}_{b_{k}b_{i}}}={\hat{R}}_{b_{k+1}b_{k}},\frac{\partial {\tilde{\theta }}_{b_{k+1}b_{i}}}{\partial n_{r}}={\hat{R}}_{b_{k+1}b_{k}}\frac{J_{r}(-\frac{d_{r}-d_{l}}{d})}{d},\frac{\partial {\tilde{\theta }}_{b_{k+1}b_{i}}}{\partial n_{l}}=-{\hat{R}}_{b_{k+1}b_{k}}\frac{J_{r}(-\frac{d_{r}-d_{l}}{d})}{d}$$

Similarly, the error transfer equation in the translation direction can be obtained as:

(25) p˜bibk+1=p˜bibk+R^bkbiTRbkbk’([Jr(Δθ2)nr−nl2d]×Δbks^bk+1bk+nr−nl2)+R^bkbiT[θ˜bkbi]×Rbkbk’Δbks^bk+1bk

The error transfer coefficient can be calculated as:

(26) ∂p˜bibk+1∂p˜bibk=I3,∂p˜bibk+1∂θ˜bkbi=−R^bkbiT[Rbkbk’Δbks^bk+1bk]×∂p˜bibk+1∂nr=R^bkbiTRbkbk’(12I3−[Δbks^bk+1bk]×Jr(Δθ2)12d)∂p˜bibk+1∂nr=R^bkbiTRbkbk’(−12I3+[Δbks^bk+1bk]×Jr(Δθ2)12d)

The wheel odometer error transfer calculation can then be performed according to Equation (7). In the mobile robot system, vision information can be used to detect whether the wheel odometer is slipping. According to the positional relationship RCO and PCO between the camera and the wheel odometer coordinate frame, the relative movement RCjCi and PCjCi of the rotation and position of the camera can be calculated. Then, the essential matrix E=[PCjCi]×RCjCi between two frames can be constructed. According to the camera internal parameter *K*, we can calculate the essential matrix $F=K^{-T}EK^{-1}$. Assume that the corresponding feature point observations of image frames *i* and *j* are $P_{1}={\left(u_{1},v_{1},1\right)}^{T}$ and $P_{2}={\left(u_{2},v_{2},1\right)}^{T}$. The distance from $P_{1}$ to the corresponding epipolar line can then be calculated as:

(27) $$D_{P_{1}P_{2}}=\frac{P_{1}^{T}FP_{2}}{\sqrt{{\left(FP_{2}\right)}_{x}^{2}+{\left(FP_{2}\right)}_{y}^{2}}}$$

According to the threshold th, count the number of feature points with $D_{P_{1}P_{2}}<th$ and calculate the ratio to the total number of feature points. If the ratio exceeds a certain threshold, the wheel odometer is considered to be slipping. If there is no slip, the state in the sliding window can be updated by using the pre-integration component measured by the wheel odometer, which is similar to the method of updating by using IMU pre-integration. In addition, note that the state variables in the sliding window are defined in the IMU coordinate frame, so the extrinsic parameters from the wheel odom coordinate frame to the IMU coordinate frame need to be used during the update process to convert $R_{b_{j}b_{i}}$ and $p_{b_{i}b_{j}}$ in the wheel odom coordinate frame to the IMU coordinate system.

### 2.5. Helmert Variance Component Estimation

In the update process of the system proposed in this article, because it involves visual feature points, IMU pre-integration, and observation information from the wheel odometer, the determination of the weight between observations is very important. However, their weights are generally inappropriate because of the errors of parameter calibration and calculation and the corresponding unit weight variances. Taking observations of visual feature points and IMU pre-integration as an example, the update equation of the observations to the state variables of the system based on extended Kalman filter is:

(28) $$\begin{array}{l} \hat{X}=\bar{X}+K(Z_{m}-H_{m}\bar{X})=\bar{X}+Kr_{m}\\ {\hat{N}}^{-1}=(I-KH_{m}){\bar{N}}^{-1}{\left(I-KH_{m}\right)}^{T}+KR_{m}K^{T}\\ K={\bar{N}}^{-1}H_{m}^{T}{\left(H_{m}{\bar{N}}^{-1}H_{m}^{T}+R_{m}\right)}^{-1} \end{array}$$

where *m = f* or *I*, which indicate that the system state is updated by visual feature points and IMU pre-integration, respectively. $\bar{X}$ and $\hat{X}$ represent the state variables of the system before and after the update; ${\bar{N}}^{-1}$ and ${\hat{N}}^{-1}$ are the covariance matrices of the system state vector variables before and after the update; $Z_{m}$ is the observation and $R_{m}$ is corresponding covariance matrix; $H_{m}$ is the Jacobian matrix of the observed measurement to the system state vector variables, and its definition is the same as Equations (12) and (17); K is the gain matrix and I is the identity matrix; $r_{m}$ can be calculated by Equations (12) and (17). Then, the calculation equation of weight can be obtained by Helmert variance component estimation theory [18,20] as:

(29) $$\left[\begin{array}{l} r_{f}R_{f}^{-1}r_{f}\\ r_{I}R_{I}^{-1}r_{I} \end{array}\right]=\left[\begin{array}{cc} n_{1}-tr(N_{f}N^{-1})+tr(N_{f}N^{-1}N_{f}N^{-1}) & tr(N_{f}N^{-1}N_{I}N^{-1})\\ tr(N_{f}N^{-1}N_{I}N^{-1}) & n_{2}-tr(N_{I}N^{-1})+tr(N_{I}N^{-1}N_{I}N^{-1}) \end{array}\right]\left[\begin{array}{l} \sigma_{1}^{2}\\ \sigma_{2}^{2} \end{array}\right]$$

where $N_{f}=H_{f}^{T}R_{f}^{-1}H_{f}$ and $N_{I}=H_{I}^{T}R_{I}^{-1}H_{I}$. $R_{f}$ and $R_{I}$ are the covariance of visual feature points and pre-integration observations, respectively. The “tr” item represents the trace of the matrix. Because it is much smaller than the observation numbers $n_{1}$ and $n_{2}$ in the sliding window calculation process, the trace items can be ignored in the calculation to improve the calculation efficiency. Using Equation (29), $\sigma_{1}^{2}$ and $\sigma_{2}^{2}$ can be calculated. Furthermore, the covariance matrices ${\bar{R}}_{f}=\frac{\sigma_{1}^{2}}{\sigma_{0}^{2}}R_{f}$ and then ${\bar{R}}_{I}=\frac{\sigma_{2}^{2}}{\sigma_{0}^{2}}R_{I}$ can be updated, where $\sigma_{0}^{2}$ is an arbitrary constant and the unit weight variance $\sigma_{f}^{2}$ of the feature point can be taken here. The system state vector variables and their covariance can be updated directly using Equation (28) after ${\bar{R}}_{f}$ and ${\bar{R}}_{I}$ are determined.

## 3. Simulations

### 3.1. Simulation Environment Settings

**Figure 2:**
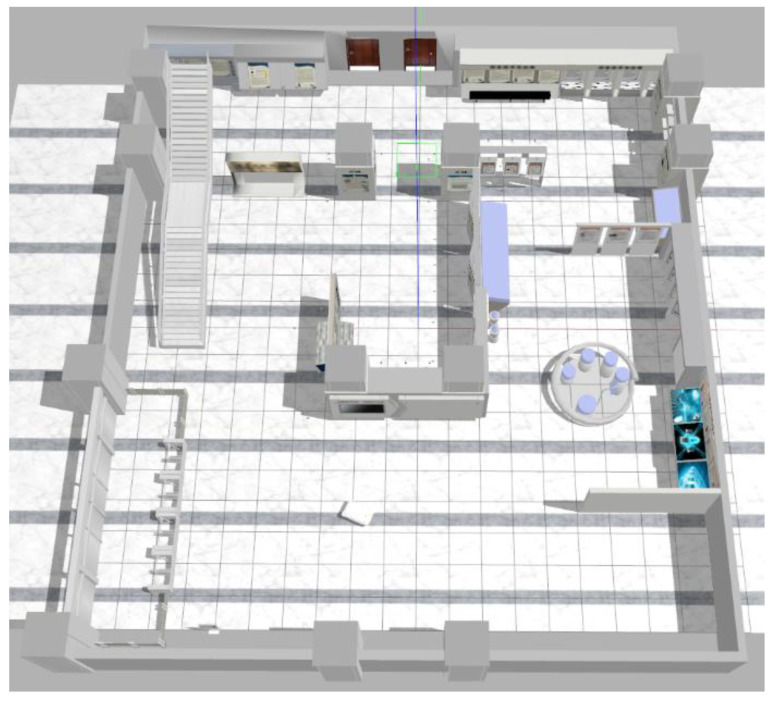
The Gazebo simulation environment.

To verify the effect of the algorithm proposed in this article on the localization accuracy, we first conduct a simulation analysis of the algorithm in a simulation environment built in Gazebo. We directly use the simulation environment provided by the Chinese Academy of Sciences [28], which contains rich environmental feature information, and import the Mir robot URDF model, as shown in Figure 2. Mir is a differential drive robot equipped with a camera, IMU, and wheel odometer. The camera can output images with a resolution of 1080 × 1920 at a rate of 10 Hz, and the IMU outputs acceleration and angular velocity information at 150 Hz. The wheel odometer outputs speed information and angular velocity information at 50 Hz. During the simulation process, the maximum linear velocity and maximum angular velocity of the robot motion are 1.0 m/s and 0.7 rad/s, respectively. Sensor data are sent and received in the form of ROS (Robot Operating System) topics. The computer is configured with Intel Core i7-7700K CPU with 3.5 GHz, 16 GB RAM, and the system is Ubuntu 18.04 and ROS Melodic.

### 3.2. Analysis of Localization Accuracy

**Figure 3:**
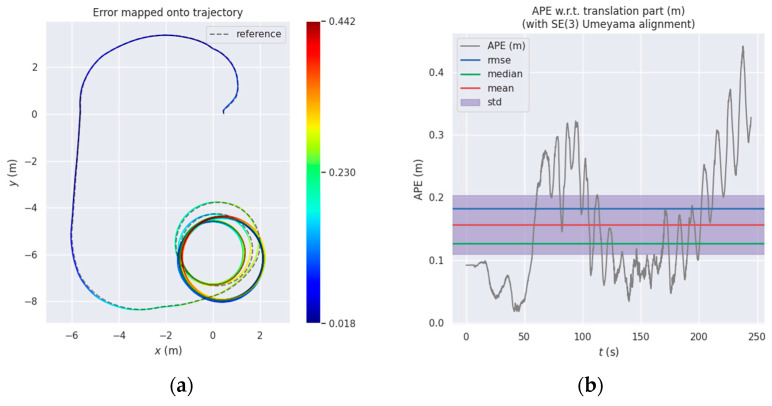

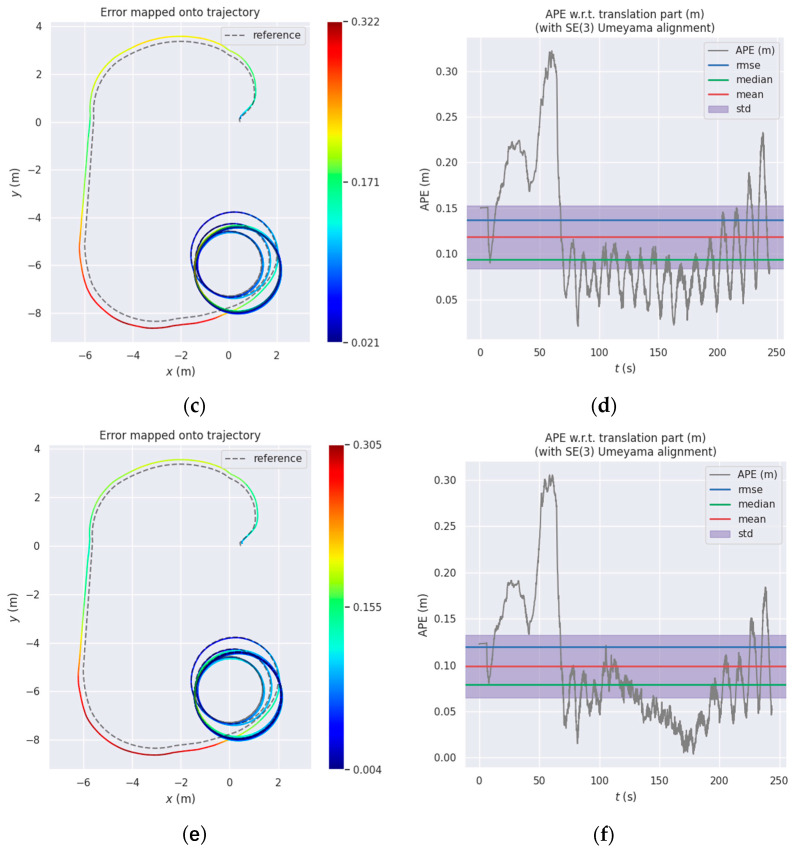
Comparison of the proposed method versus OpenVINS: (**a**) OpenVINS; (**c**) OpenVINS+IMU Pre-integration; (**e**) OpenVINS+IMU Pre-integration + Odom; (**b**,**d**,**f**) are the corresponding errors.

The following is the analysis of the trajectory estimation accuracy of the algorithm proposed in this paper and the original OpenVINS trajectory estimation accuracy of the robot in two motion situations, that is, the motion mainly composed of uniform circular motion or uniform linear motion. All algorithm tests are based on stereovision fusion with IMU and wheel odometer information. We use the open source tool evo [29] and use absolute pose error (APE) as the error evaluation standard to analyze the accuracy of the trajectory. Figure 3a,c,e shows several comparison heat maps of predicted trajectory and true trajectory (without alignment and scale correction). The motion of the robot is mainly composed of uniform circular motion. The redder the color in the figure, the greater the translation error. The corresponding errors are shown in Figure 3b,d,f.

Table 1 shows statistics of trajectory RMSE (Root Mean Squared Error) of translation and rotation. In circular motion, the stereo VIO system will not increase the trajectory estimation error sharply as does the monocular VIO system. Because the stereo can provide the system with a certain scale of observability, therefore, the RMSE of the translation during its circular motion is not very large, which is only 0.1826 m. After introducing the IMU pre-integration as the observation and using the HVCE method to obtain the maximum a posteriori weight of the covariance between the visual observation and the IMU pre-integration, using this constraint to update the system state can reduce the RMSE to 0.1368 m and improve the accuracy by 25.08%. If the system introduces wheel odometer information at the same time, the error can be further reduced which the RMSE of the translation is reduced to 0.1197 m and the accuracy is improved by 34.45%. This fully proves that the algorithm proposed in this paper has a positive effect on localization accuracy after introducing pre-integration information.

**Figure 4:**
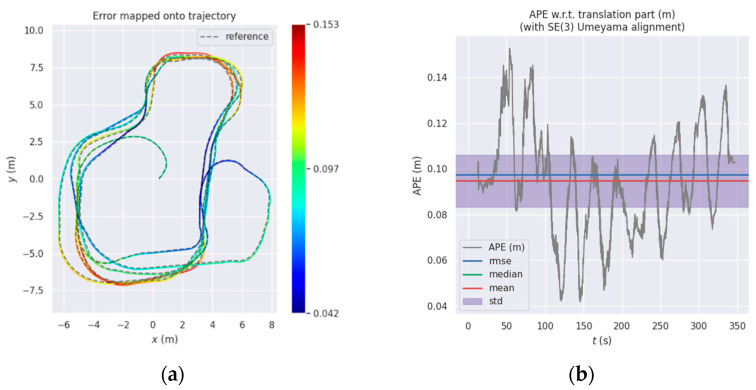

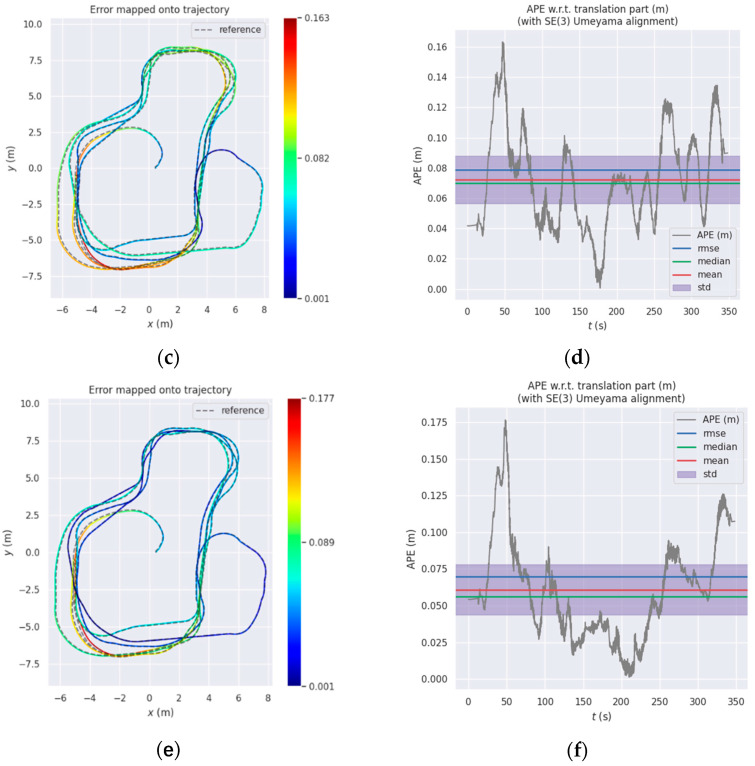
Comparison of the proposed method versus OpenVINS: (**a**) OpenVINS; (**c**) OpenVINS+IMU Pre-integration; (**e**) OpenVINS+IMU Pre-integration +Odom; (**b**,**d**,**f**) are the corresponding errors.

When the robot is mainly in uniform linear motion and in a closed loop, the heat maps of the estimated trajectory of the algorithm and the real trajectory are shown in Figure 4a,c,e and the corresponding errors are shown in Figure 4b,d,f. The statistical results of the RMSE of translation and rotation are shown in Table 2. The RMSE of the translation part of the original OpenVINS algorithm is 0.1011 m. After using the IMU pre-integration as the observation constraint and obtaining the optimal a posterior weight to update the state of the system by HVCE method, the RMSE of translation of the system can be reduced to 0.0787 m, and the position accuracy can be improved by 22.15%. Because the robot moves on a plane in the simulation environment, and the wheel odometer pre-integration can provide such constraints, the positioning error can be further reduced to 0.0698 m, and the position accuracy can be improved by 30.95%. It can be seen that in both cases the robot’s motion error is relatively small, mainly because the robot’s motion speed in the simulation environment is low (maximum 0.8 m/s) and stable, so the error is relatively small. However, in actual situations, especially in the process of autonomous navigation, there are situations where the speed and angular velocity change drastically which may cause error increased rapidly in the absence of loopback. Therefore, next we conduct experiments on the actual data collected and the actual robot to verify and evaluate the performance of the algorithm in real world scenarios.

## 4. Experiments

### 4.1. Public Dataset Test

To verify the effectiveness of the algorithm, we tested it on the EuRoc MAV Dataset. The EuRoc dataset includes the global shutter camera and Micro Electro Mechanical Systems (MEMS) IMU configured on the drone to simultaneously collect stereoimages and IMU data. The true value of the drone’s motion trajectory is provided through the motion capture system. The collection environment of the dataset includes three environments, one is the machine hall and the other two are the Vicon room. The algorithm proposed in this paper compared the performance of trajectory estimation with three classical VIO methods which include S-MSCKF, OpenVINS, and VINS-Fusion without loop closure. S-MSCKF supports stereo and OpenVINS supports monocular and stereo mode. They are both VIO methods that tightly couple visual information and IMU measurements with extended kalman filters. VINS-Fusion is an optimization method which supports a monocular and stereo visual-inertial navigation system and uses tight coupling of visual measurement and pre-integration in a sliding window.

In the experiments, we use the open source tool evo to evaluate the accuracy of the results of the algorithm running on the dataset and use Absolute Pose Error (APE) as the error evaluation standard. Table 3 lists the Root Mean Square Errors (RMSE) of translation and rotation of the estimated trajectory of the proposed method and the state-of-art stereo visual-inertial navigation system. From the table, it can be seen intuitively that OpenVINS gives better positioning performance than the other two mainstream methods by adopting the strategies of using FEJ, adding camera intrinsic parameters, IMU and camera extrinsic parameters, time drift between IMU and camera, and SLAM features to the state vector. Our algorithm adds pre-integration constraints to the basic framework of OpenVINS as observations, which can also further improve the positioning accuracy of the system. Especially when the dataset contains long linear motion, which is similar to the motion of indoor mobile robot, the algorithm proposed in this paper improves the positioning accuracy more obviously. For example, for the OpenVINS system with excellent positioning performance, in the MH-04-difficult and MH-05-difficult sequence, the RMSE of the translation is reduced from 0.1625 m and 0.1518 m to 0.1162 m and 0.1031 m, a reduction of 28.49% and 32.08%, respectively. The symbol “×” indicates that the dataset could not be completed in the program.

**Figure 5:**
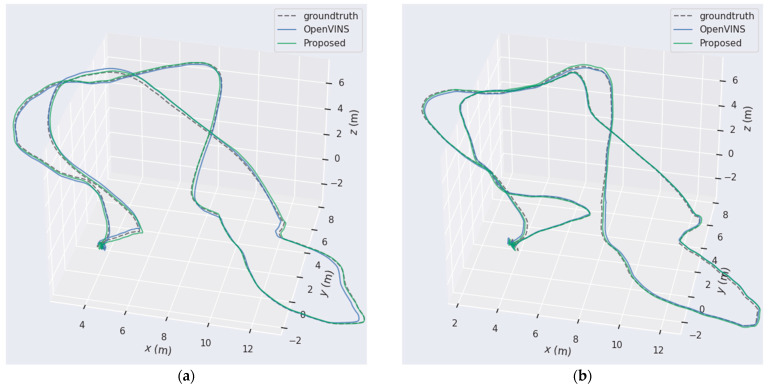
The comparison of estimated trajectory between OpenVINS and the proposed method for the (**a**) MH-04-difficult and (**b**) MH-05-difficult sequences.

To display the estimation results intuitively, Figure 5 shows the comparison of trajectory estimation between the original OpenVINS and the algorithm proposed in this paper in the MH-04-difficult and MH-05-difficult sequences. This sequence involves the drone entering a dark environment; it is difficult to extract feature points and the probability of mismatching increases. Only using visual features to update the system status leads to larger errors. Here, the pre-integration of the IMU, which is not affected by light, is introduced as an observation to update the status of the system, which can improve the positioning accuracy of the system.

### 4.2. Real-World Test

**Figure 6:**
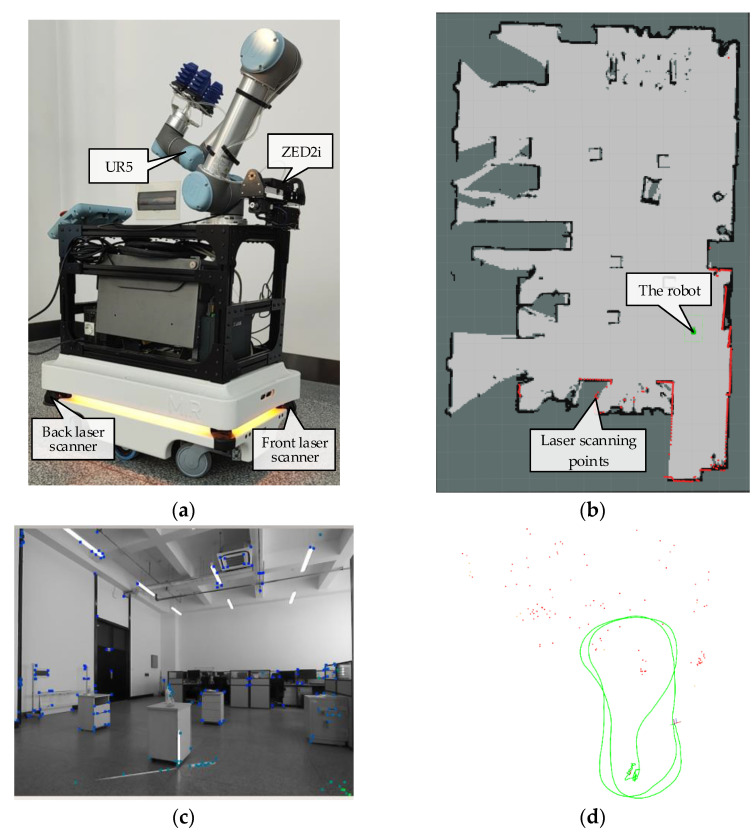
(**a**) The experiment platform; (**b**) localization of the robot on the established environment map; (**c**) camera view (the blue points are SLAM tracking points); (**d**) estimated trajectory of the robot (red points indicate SLAM feature points).

To verify the performance of the algorithm in real-world scenarios, the self-built Mir robot mobile platform is used for experiments, as shown in Figure 6a. The Mir robot is equipped with front and back laser sensors and wheel odometer. At the same time, the platform is also equipped with a ZED2i camera produced by STEREOLABS, which can output the stereoimage and IMU message required by the algorithm described in this paper. The image accepted by the algorithm is a three-channel color image and is first converted into a single-channel grayscale image with 720 × 1280 resolution and 20 Hz frequency. In this experiment, the cartographer algorithm [30] is used to build the environment map through the laser sensors. Since the loop closure can be used to eliminate the cumulative error of the map in the process of map building, it can achieve a high map-building accuracy. During the movement of the robot, the adaptive Monte Carlo localization algorithm is used, which applies the acquired laser scanning information and the established environment map to obtain globally consistent positioning information. The localization of the robot on the map is shown in Figure 6b, and its positioning accuracy reaches 5 cm, which meets the requirements of the visual–inertial odometry (without closed loop) accuracy evaluation benchmark. The test trajectory of the robot is a closed-loop motion in an indoor environment. The feature point tracking and the estimated trajectory displayed in RVIZ during motion are shown in Figure 6c,d, respectively.

**Figure 7:**
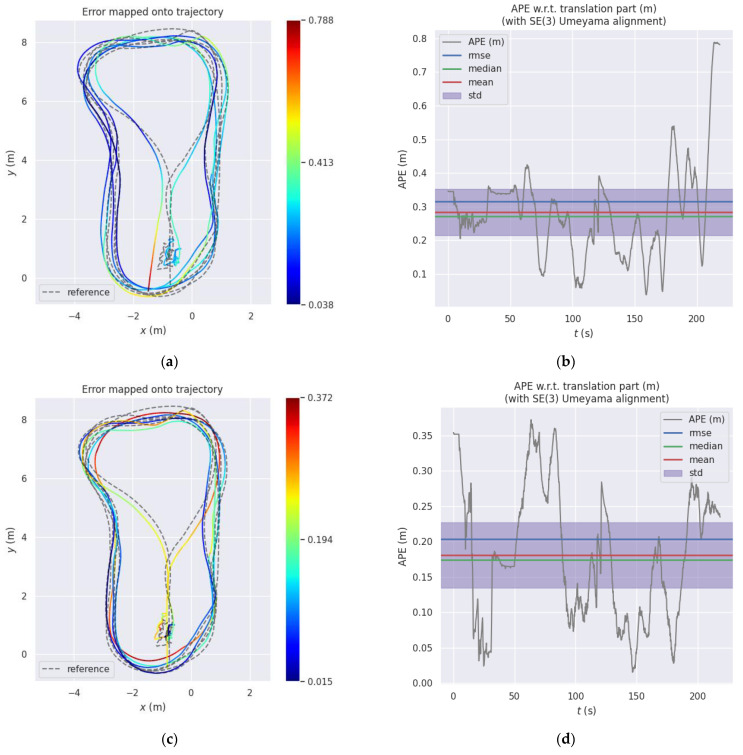

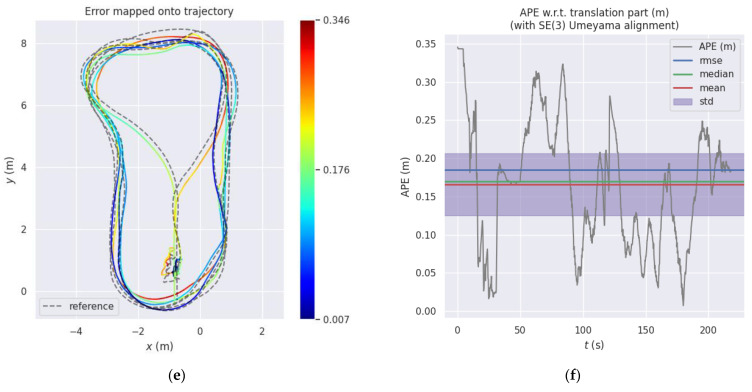
Comparison of the proposed method versus OpenVINS: (**a**) OpenVINS; (**c**) OpenVINS+IMU Pre-integration; (**e**) OpenVINS+IMU Pre-integration +Odom; (**b**,**d**,**f**) are the corresponding errors.

The trajectory length of the entire test is about 122 m, and the maximum linear velocity and maximum angular velocity are 1.0 m/s and 0.7 rad/s, respectively, during robot motion. As with the above method for accuracy analysis, we continue to use evo_ape for trajectory accuracy analysis, as shown in Figure 7. Figure 7a,c,e shows the comparison of the estimated trajectory with the ground truth, and Figure 6b and Figure 7d,f indicate the corresponding errors. The RMSE of translation is counted separately, the error of the original OpenVINS algorithm is 0.3145 m, the trajectory error after constraint update using IMU pre-integration is 0.2132 m, and the error after further using wheel odometer pre-integration update is 0.1841 m. It can be seen that adding IMU pre-integration constraints can greatly improve the positioning accuracy of the system, and the accuracy is increased by 32.21% compared with the original OpenVINS method. Furthermore, by adding the wheel odometer as another observation constraint, the accuracy can be further improved by 41.46%.

## 5. Discussion

In this paper, in order to make full use of the sensor information to improve the positioning accuracy of the system, three main works were carried out in the VIO system based on the MSCKF framework. The first was to use the pre-integration of the IMU as the observation information of the system, and use the standard EKF method to update the state of the system. Secondly, in the positioning process for indoor mobile robots, we used the same idea to extract the pre-integrated information of the wheel odometer, and constrain and update the state of the system. In addition, visual features are collected and used as observations to update the system state in the MSCKF framework. This paper uses the Helmert variance component estimation method to determine a more reasonable covariance weight between visual features and IMU pre-integration. To verify the effectiveness of the algorithm proposed in this paper, we tested it in the Gazebo simulation environment, public dataset, and actual environment. In the Gazebo simulation environment, the accuracy analysis was carried out on the situation where the robot mainly moved in a circular motion or in a uniform linear motion. The results show that the algorithm proposed in this paper has a significant improvement in accuracy compared to the OpenVINS algorithm. In the case of using IMU pre-integration with the HVCE method, the positioning accuracy of translation can be improved by 25.08% and 22.15% in two cases, respectively. Meanwhile, if the pre-integration information of the wheel odometer is used, the accuracy can be further improved by 34.45% and 30.95%. On the public dataset, we compared the positioning accuracy performance of the proposed algorithm with several mainstream algorithms: S-MSCKF, VINS-Fusion, and OpenVINS. All test results show the excellent performance of the algorithm proposed in this paper. Especially in a complex environment, such as the MH-04-difficult and MH-05-difficult sequences, due to obvious changes in illumination, it has a greater impact on visual measurement. Compared with OpenVINS, the algorithm proposed in this paper can reduce the RMSE of translation from 0.1625 m and 0.1518 m to 0.1162 m and 0.1031 m and increase the accuracy by 28.49% and 32.08%. Finally, we also conducted the corresponding accuracy analysis experiments in the actual robot, and proved that the proposed algorithm can improve the accuracy by 32.21% compared with OpenVINS after introducing IMU pre-integration as the observation constraint. Similarly, after we introduce the pre-integration of the wheel odometer as the observation constraint, the accuracy of the system can be further enhanced, which is 41.46% higher than the original OpenVINS. However, in our actual experimental verification process, we also found that the parameters of the IMU, including the noise variance of the accelerometer and the gyroscope, and the variance of the random walk noise, have a greater impact on the performance of the system. Even if the parameter selection is inappropriate, it may cause the system to crash. In the next step, we will conduct further research on how to improve the robustness of the system to parameters. There are also problems including the estimation of external parameters between the wheel odometer and the camera while using the wheel odometer. The time stamp between the two sensors are not strictly hardware aligned, which will be further solved in the future. 

## 6. Conclusions

In this paper, the pre-integration of the IMU is introduced as an observation, and the state vector in the sliding window is updated, making full use of the information of the IMU and improving the system positioning accuracy in the MSCKF framework. At the same time, since the system also involves the observation of visual feature points, in order to obtain the appropriate covariance between the two observations, this paper uses the Helmert variance component estimation method to estimate the maximum a posterior weight of the covariance. For indoor mobile robots, the same method is used, and the pre-integration of the wheel odometer is used as the constraint of the pose estimation, which further improves the positioning accuracy of the system. Additionally, through the Gazebo environment simulation, public dataset EuRoc, and actual experiments the performance of the proposed algorithm is verified. Analysis of the results leads to following conclusions. One is that introducing pre-integration by IMU and wheel odometer as observation constraints in MSCKF framework can help improve the positioning accuracy of the system. The other one is that with the Helmert variance component estimation method, the largest a posteriori covariance weight between visual observation and pre-integration observation can be obtained. This method can further improve the positioning accuracy of the system. Since the wheel odometer can only provide plane constraint information, namely x, y, yaw directions, and in an indoor environment, the ground is not completely flat. In the next task, we will model the ground more accurately so that higher localization accuracy can be obtained after using the wheel odometer information.

## Figures and Tables

**Table 1:** The Root Mean Square Error (RMSE) results of OpenVINS and the proposed algorithm.

Evaluation	OpenVINS		OpenVINS + IMU + HVCE		OpenVINS + IMU + Odom + HVCE	
RMSE	Trans (m)	Rot (°)	Trans (m)	Rot (°)	Trans (m)	Rot (°)
	0.1826	2.1098	0.1368	1.6815	0.1197	0.7797
Improvement			25.08%	20.30%	34.45%	63.04%

**Table 2:** The Root Mean Square Error (RMSE) results of OpenVINS and the proposed algorithm.

Evaluation	OpenVINS		OpenVINS + IMU + HVCE		OpenVINS + IMU + Odom + HVCE	
RMSE	Trans (m)	Rot (°)	Trans (m)	Rot (°)	Trans (m)	Rot (°)
	0.1011	0.7536	0.0787	0.5475	0.0698	0.3743
Improvement			22.15%	27.34%	30.95%	50.33%

**Table 3:** The Root Mean Square Error (RMSE) results of OpenVINS and the proposed algorithm.

Seq	S-MSCKF		VINS-Fusion		OpenVINS		The Proposed	
	Trans (m)	Rot (°)	Trans (m)	Rot (°)	Trans (m)	Rot (°)	Trans (m)	Rot (°)
V1_02_medium	0.1082	2.4125	×	×	0.0542	1.8723	0.0480	1.8564
V1_03_difficult	0.1654	4.1323	0.1076	6.8387	0.0516	2.5557	0.0512	2.3123
V2_02_medium	0.1174	1.7794	0.1167	2.8392	0.0462	1.4552	0.0469	1.3057
V2_03_medium	×	×	×	×	0.0708	0.9819	0.0601	0.8100
MH_03_medium	0.2889	2.0835	0.2856	1.4097	0.1079	1.3833	0.0980	1.3748
MH_04_difficult	0.2804	1.1874	0.4241	2.3703	0.1625	1.2023	0.1162	1.0628
MH_05_difficult	0.4001	1.1348	0.3081	1.7703	0.1518	1.2390	0.1031	0.9418

## Data Availability

All the data generated during the experiments are presented in the article.

## Appendix A

We define the IMU error state vector and the noise vector at time *k* as ${\left[\begin{array}{ccccc} {\tilde{\theta }}_{b_{k}} & {\tilde{\alpha }}_{b_{k}} & {\tilde{\beta }}_{b_{k}} & {\tilde{b}}_{k}^{g} & {\tilde{b}}_{k}^{a} \end{array}\right]}^{T}$ and ${\left[\begin{array}{cccccc} n_{k}^{g} & n_{k}^{a} & n_{k+1}^{g} & n_{k+1}^{a} & n_{b_{k}^{g}} & n_{b_{k}^{a}} \end{array}\right]}^{T}$, respectively. Then the IMU error state vector at time *k* + 1 can be expressed by the error state propagation equation as:

(A1) $$\left[\begin{array}{l} {\tilde{\theta }}_{b_{k+1}}\\ {\tilde{\alpha }}_{b_{k+1}}\\ {\tilde{\beta }}_{b_{k+1}}\\ {\tilde{b}}_{k+1}^{a}\\ {\tilde{b}}_{k+1}^{g} \end{array}\right]=F\left[\begin{array}{l} {\tilde{\theta }}_{b_{k}}\\ {\tilde{\alpha }}_{b_{k}}\\ {\tilde{\beta }}_{b_{k}}\\ {\tilde{b}}_{k}^{g}\\ {\tilde{b}}_{k}^{a} \end{array}\right]+G\left[\begin{array}{l} n_{k}^{g}\\ n_{k}^{a}\\ n_{k+1}^{g}\\ n_{k+1}^{a}\\ n_{b_{k}^{g}}\\ n_{b_{k}^{a}} \end{array}\right]$$

with

(A2) $$\begin{array}{l} F=\left[\begin{array}{ccccc} {\hat{R}}_{b_{k+1}b_{k}} & 0 & 0 & -{\hat{R}}_{b_{k+1}b_{k}}J_{r}(-{\left[\hat{w}\Delta t\right]}_{\times }) & 0\\ f_{21} & I & I\Delta t & f_{24} & -\frac{1}{4}(R_{b_{i}b_{k}}+R_{b_{i}b_{k+1}})\Delta t^{2}\\ f_{31} & 0 & I & f_{34} & -\frac{1}{2}(R_{b_{i}b_{k}}+R_{b_{i}b_{k+1}})\Delta t\\ 0 & 0 & 0 & I & 0\\ 0 & 0 & 0 & 0 & I \end{array}\right]\\ G=\left[\begin{array}{cccccc} \frac{1}{2}I\delta t & 0 & \frac{1}{2}I\delta t & 0 & 0 & 0\\ g_{21} & \frac{1}{4}R_{b_{i}b_{k}}(-\Delta t^{2}) & g_{23} & \frac{1}{4}R_{b_{i}b_{k+1}}(-\Delta t^{2}) & 0 & 0\\ g_{31} & -\frac{1}{2}R_{b_{i}b_{k}}\Delta t & g_{33} & -\frac{1}{2}R_{b_{i}b_{k+1}}\Delta t & 0 & 0\\ 0 & 0 & 0 & 0 & I & 0\\ 0 & 0 & 0 & 0 & 0 & I \end{array}\right] \end{array}$$

(A3) $$\begin{array}{l} f_{21}=-\frac{1}{4}R_{b_{i}b_{k}}{\left[{\hat{a}}^{b_{k}}\Delta t^{2}\right]}_{\times }-\frac{1}{4}R_{b_{i}b_{k+1}}{\left[{\hat{a}}^{b_{k+1}}\Delta t^{2}\right]}_{\times }\text{​​​}\\ f_{24}=\frac{1}{4}{\left[R_{b_{i}b_{k+1}}{\hat{a}}^{b_{k+1}}\right]}_{\times }\Delta t^{2}{\hat{R}}_{b_{k+1}b_{k}}J_{r}(-{\left[\hat{w}\Delta t\right]}_{\times })\\ f_{31}=-\frac{1}{2}R_{b_{i}b_{k}}{\left[{\hat{a}}^{b_{k}}\Delta t\right]}_{\times }-\frac{1}{2}R_{b_{i}b_{k+1}}{\left[{\hat{a}}^{b_{k}+1}\Delta t\right]}_{\times }{\hat{R}}_{b_{k+1}b_{k}}\\ f_{34}=\frac{1}{2}R_{b_{i}b_{k+1}}{\left[{\hat{a}}^{b_{k}+1}\Delta t\right]}_{\times }{\hat{R}}_{b_{k+1}b_{k}}J_{r}(-{\left[\hat{w}\Delta t\right]}_{\times })\Delta t\\ g_{21}=\frac{1}{4}R_{b_{i}b_{k+1}}{\left[{\hat{a}}^{b_{k+1}}\Delta t^{2}\right]}_{\times }{\hat{R}}_{b_{k+1}b_{k}}J_{r}(-{\left[\hat{w}\Delta t\right]}_{\times })\frac{\Delta t}{2}\\ g_{23}=\frac{1}{4}R_{b_{i}b_{k+1}}{\left[{\hat{a}}^{b_{k+1}}\right]}_{\times }\Delta t^{2}{\hat{R}}_{b_{k+1}b_{k}}J_{r}(-{\left[\hat{w}\Delta t\right]}_{\times })\frac{\Delta t}{2}\\ g_{31}=\frac{1}{2}R_{b_{i}b_{k+1}}{\left[{\hat{a}}^{b_{k}+1}\Delta t\right]}_{\times }{\hat{R}}_{b_{k+1}b_{k}}J_{r}(-{\left[\hat{w}\Delta t\right]}_{\times })\frac{\Delta t}{2}\\ g_{33}=\frac{1}{2}R_{b_{i}b_{k+1}}{\left[{\hat{a}}^{b_{k}+1}\Delta t\right]}_{\times }{\hat{R}}_{b_{k+1}b_{k}}J_{r}(-{\left[\hat{w}\Delta t\right]}_{\times })\frac{\Delta t}{2} \end{array}$$

where $J_{r}(\cdot )$ is the right Jacobian of **SO**(3) that maps the variation in rotation angle in the parameter vector space into the variation in the tangent vector space to the manifold. The covariance of the pre-integration can be calculated iteratively according to the Equation (A1):

(A4) $$\sum_{b_{i}b_{k+1}}=F\sum_{b_{i}b_{k+1}}F^{T}+GQ_{d}G^{T}$$

The covariance matrix at the initial iteration is $\sum_{b_{i}b_{k}}=I$ and $Q_{d}$ is the covariance of the IMU raw measurements. Meanwhile, the Jacobian matrix of the pre-integration with respect to the error state can be directly iteratively calculated $J_{ik+1}=FJ_{ik}$ with $J_{ii}=I$. This Jacobian matrix can be used in Equation (10) to complete the pre-integration update due to bias changes.
